# Association of vitamin D receptor gene variants with polycystic ovary syndrome: a meta-analysis

**DOI:** 10.1186/s12881-019-0763-5

**Published:** 2019-02-14

**Authors:** Xiao-Yuan Shi, Ai-Ping Huang, Duo-Wen Xie, Xiao-Long Yu

**Affiliations:** 1 Lanzhou University Second Hospital, Medical Record Department, Lanzhou, 730030 Gansu Province China; 2 Blood Center of Zhejiang Province, Blood Donation Service Department, Hangzhou, 310006 Zhejiang Province China; 3 Lanzhou University Second Hospital, Intensive Care Unit 2, Lanzhou, 730030 Gansu Province China; 4 Zhejiang Hospital, Department of nutrition, Hangzhou, 310013 Zhejiang Province China

**Keywords:** Polycystic ovary syndrome, Vitamin D receptor, Polymorphisms, Meta-analysis

## Abstract

**Background:**

Polycystic ovary syndrome (PCOS) is a common endocrine disorder in reproductive-age women. Multiple susceptible gene as well as environmental factors and their interaction each other are contributed to the PCOS risk. Several case-control studies have researched the associations of the *vitamin D receptor* gene (*VDR*) polymorphisms with PCOS susceptibility, but the jury is still out. Here, we carried out a meta-analysis to clarify polymorphisms between *ApaI (C/A) (rs7975232), BsmI (G/A) (rs1544410), FokI (C/T) (rs10735810), TaqI (T/C) (rs731236) and Tru9I (G/A) (rs757343)* in the *VDR* gene and PCOS susceptibility based on relative lager sample size.

**Methods:**

English database of PubMed and Embase, and Chinese database of Wanfang and China National Knowledge Infrastructure (CNKI) databases were retrivaled for the relationship between *VDR* gene variates and PCOS susceptibility published before 31th, May 2018. Crude odds ratios (ORs) and its 95% confidence intervals (95% CIs) in different comparisons were used to detected the strength of the association. All the statistical analyses of the present meta-analysis were performed by STATA version 12.0 software.

**Results:**

Totally, 3587 (PCOS group 1922; control group 1665) participants from 13 studies were included which met our inclusion criteria. A statistically significant association between *VDR ApaI (rs7975232)* polymorphism and PCOS susceptibility (C vs. A: OR = 1.19, 95%CI = 1.06~1.34, *P* = 0.004) was found in the overall population. After stratified by ethnicity, we showed that there is a significant association between *VDR ApaI (rs7975232)* polymorphism and susceptibility to PCOS in the Asian (C vs. A: OR = 1.21, 95%CI = 1.04~1.42, *P* = 0.016) population, but this association was not found in the Caucasian population. Additionally, a significant relationship between *VDR BsmI (rs1544410)* variates with PCOS susceptibility in the Asian (G vs. A: OR = 1.27, 95%CI = 1.06~1.53, *P* = 0.011) population, but this association was not found in the Caucasian population. We didn’t find any association between *VDR FokI (rs2228570), VDR TaqI (rs731236), VDR Tru9I (rs757343)* and PCOS susceptibility in the overall and the subgroup populations.

**Conclusions:**

Our findings demonstrated that *VDR ApaI (rs7975232) and VDR BsmI (rs1544410)* polymorphisms are correlated with susceptibility to PCOS in the Asian population and *VDR TaqI (rs731236), VDR FokI (rs2228570), VDR Tru9I (rs757343)* did not reveal a relationship with the PCOS susceptibility.

## Background

A common endocrine syndrome, polycystic ovary syndrome (PCOS), is characterized by long-term absence of ovulation and high androgen, which is the most common causes of menstrual disorders and infertility in women during reproductive years [1, 2]. The main clinical manifestations of PCOS include abnormal menstruation, ovulation disorder and infertility, hirsutism and acne [3]. In addition to the menstrual disturbance and hyperandrogenism, PCOS patients demonstrate an increased prevalence of type 2 diabetes mellitus, impaired glucose tolerance, hyperinsulinemia, insulin resistance (IR), and obesity [4, 5]. The underlying causes of PCOS are not completely known. However, being a complex heterogeneous disease, genetic and environmental factors interact with each other in polycystic ovary syndrome play an vital role in the occurrence and development of the disease [6].

IR and hyperinsulinemia are frequent metabolic abnormalities in the PCOS, evidence suggests that vitamin D levels may be linked to hormonal and metabolic disorders [7]. As a steroid hormone, vitamin D could module calcium-phosphate (Ca-P) homeostasis by its conversion into the active hormone 1, 25-dihydroxycholecalciferol in the kidneys and liver, and regulate the secretion of insulin through the role on the β-cells [8, 9]. The function of vitamin D is mediated by vitamin D receptor (VDR), a ligand-dependent transcription factor in the steroid/thyroid hormone receptor superfamily that controls the pleiotropic biological effects of vitamin D [10–12]. VDR regulates about 3% of the human genome, including genes critical to glucose metabolism, but the mechanism by which VDR regulates gene expression is unclear [13].

The *VDR* gene is located on chromosome 12cenq12 and contains 14 exons. Several single nucleotide polymorphisms (SNPs) in the VDR gene have been reported, such as *ApaI in intron 8 (C/A) (rs7975232), BsmI in intron 8 (G/A) (rs1544410), FokI in exon 2 (C/T) (rs10735810), TaqI in exon 9 (T/C) (rs731236) and Tru9I in intron 8 (G/A) (rs757343).* It has been shown that VDR polymorphisms (*ApaI, BsmI, FokI, Tru9I and TaqI*) may contribute to the PCOS susceptibility, although the findings are as yet inconclusive [14–26]. A previous meta-analysis has reported the association of *VDR* gene polymorphism with incident PCOS outcomes, but only six studies were included for analysis [27]. Currently, we performed an updated systematic review and meta-analysis to more precisely evaluate the correlation between the *VDR* gene polymorphisms and PCOS susceptibility.

## Methods

### Identification of eligible studies

PubMed, EMBASE, Chinese Wanfang, China National Knowledge Infrastructure (CNKI) and other databases were searched. The retrieval period is from establishment to May 31, 2018. The search terms and keywords are as follows: “vitamin D receptor or VDR”, “polymorphisms or variants”, and “polycystic ovarian syndrome or PCOS”. References to retrieved papers were also manually searched for other potential studies not included in the database.

### Inclusion and exclusion criteria

If the study met the following criteria, it was included in the meta-analysis: (1) study on the association of *VDR* gene with PCOS; (2) case-control study design; (3) genotype distributions were available for both cases and controls to calculate an OR and its 95%CI; (4) The diagnosis of PCOS is based on the Rotterdam criteria and the National Institute of Child Health and Human Development criteria [28, 29]. Exclusion criteria are as follows: (1) abstract, case report, editorial comment, and review; (2) repeated publication; (3) studies with insufficient genotypic data; (4) studies performed on animal models.

### Quality score assessment

The quality of the study was assessed using the Newcastle-Ottawa scale. The scale is composed of three aspects: selection, comparability and exposure, with a maximum score of 9 [30]. A total score for each study of ≤3, 4–6, ≥7 is considered to be low, medium and high quality study, respectively. Any disagreements were adjusted by a third reviewer.

### Data extraction

Two researchers independently and carefully extracted the available data from each eligible study. Information on all eligible studies is as follows: (1) surname of the fist author; (2) publication year; (3) country of origin; (4) the ethnicity of population; (5) sample size of cases and controls. Our research team addressed the differences through discussion.

### Statistical analysis

The effect sizes of the association between the *VDR* gene polymorphisms and PCOS risk were calculated using odds ratios (ORs) and its 95% confience interval (95%CI). All analyses used the allelic, recessive, and dominant genetic models. The chi-square test based on Q statistics was used to analyze the inter-study heterogeneity, which was considered to be significant when *p* value < 0.10 [31]. Heterogeneity was quantified by I^2^ test. When I^2^ was less than 50%, heterogeneity was acceptable, and the fixed effect model of mantel-haenszel method was adopted. Otherwise, the random effect model based on DerSimonian and Laird methods is adopted.

We used Begg funnel plot and Egger’s linear regression test to assess potential publication bias. The Egger’s linear regression test examines the asymmetry of funnel plot measured on the natural log scale [32]. One-way sensitivity analysis was used to assess which studies had a significant impact on the stability of the results. The OR and 95% CI were estimated by STATA version 12.0 software (STATA Corporation, College Station, TX). The *P*-value of two-sided < 0.05 was considered statistically significant.

## Results

### Characteristics of eligible studies

**Fig. 1:**
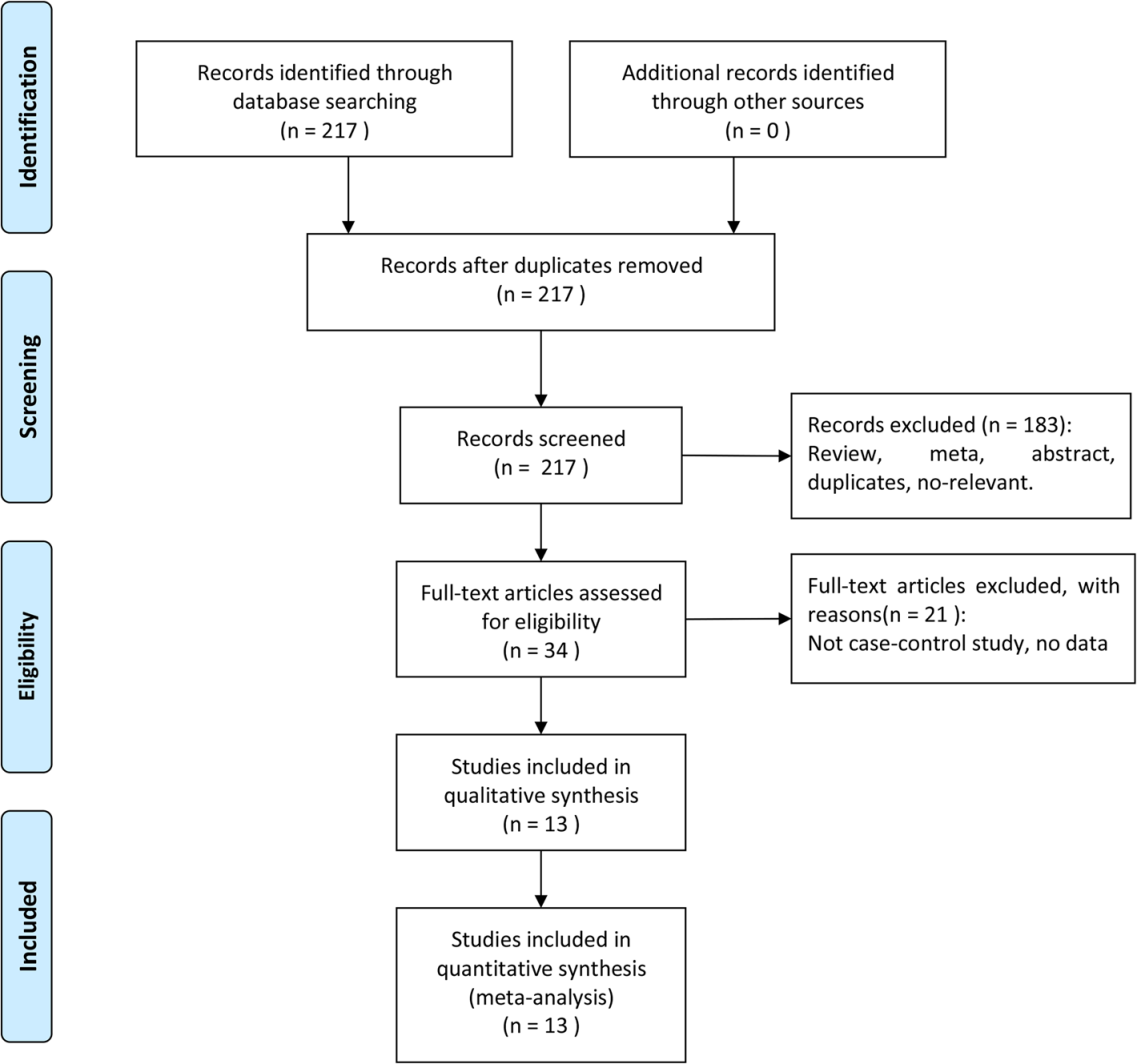
Flow chart of the literature retrieval and selection process

As Fig. 1 shows, the selection process of the studies involved in this meta-analysis was according to PRISMA flow diagram. Firstly, we searched a total of 217 articles from the above databases. Among these, 183 articles were weed out from the retrieval result due to duplicates, irrelevant topics, reviews and not about VDR gene or PCOS. Then, The remaining 34 articles were downloaded underwent full publication review carefully, we removed 21 studies because there was insufficient data to calculate OR and 95% CI and and it was not a case-control design. Finally, a total of 13 studies were included in this meta-analysis.

The association of the VDR gene *ApaI rs7975232 (G > T)* polymorphism was examined in 8 case-control studies [15–22] (Table 1), the association of the *BsmI rs1544410 (A > G)* variant was examined in 8 studies [15–17, 19–23] (Table 2), the association of the *Taq1 rs731236 (T > C)* variant was examined in 10 studies [14–22, 24] (Table 3), the association of *FokI rs2228570 (C > T)* variant was examined in 7 studies [14–17, 20, 21, 23] (Table 4) andthe association of *VDR Tru9I rs757343* with PCOS susceptibilitywas examined in 3 studies (Table 5).

**Table 1:** Characteristics of studies on VDR ApaI rs7975232 (A > C) variant and polycystic ovarian syndrome (PCOS) susceptibility

Author	Year	Country	Ethnicity	NOS scores	Sample size		Genotype in cases			Genotype in controls		
					PCOS	Control	CC	AC	AA	CC	AC	AA
Dasgupta [14]	2015	India	Asian	7	250	250	13	120	117	13	117	120
Jedrzejuk [15]	2015	Poland	Caucasian	7	90	98	19	52	19	17	49	32
Mahmoudi [16]	2015	Iran	Asian	8	35	35	9	11	15	6	21	8
Mahmoudi [17]	2009	Iran	Asian	8	162	162	36	68	58	23	90	49
El-Shal [18]	2013	Egypt	Caucasian	8	150	150	22	65	63	18	64	68
Siddamalla [19]	2017	India	Asian	7	95	130	32	21	42	25	35	70
Wehr [20]	2011	Australia	Caucasian	7	543	145	127	274	142	37	60	48
Cao [21]	2016	China	Asian	7	120	120	40	58	22	26	55	39
Santos [22]	2018	Brazil	Caucasian	7	190	100	41	88	61	16	48	36

**Table 2:** Characteristics of studies on VDR BsmI rs1544410 (A > G) variant and polycystic ovarian syndrome (PCOS) susceptibility

Author	Year	Country	Ethnicity	NOS scores	Sample size		Genotype in cases			Genotype in controls		
					PCOS	Control	GG	AG	AA	GG	AG	AA
Jedrzejuk [15]	2015	Poland	Caucasian	7	90	98	31	45	14	43	42	13
Mahmoudi [16]	2015	Iran	Asian	8	35	35	13	12	10	7	23	5
Mahmoudi [17]	2009	Iran	Asian	8	162	162	53	85	24	53	91	18
Bagheri [23]	2012	Iran	Asian	7	46	46	4	27	15	2	24	20
Siddamalla [19]	2017	India	Asian	7	94	130	34	45	15	17	41	72
Wehr [20]	2011	Australia	Caucasian	7	537	137	77	244	216	49	66	22
Cao [21]	2016	China	Asian	7	120	120	37	60	23	25	55	40
Santos [22]	2018	Brazil	Caucasian	7	187	100	74	76	37	41	48	11

**Table 3:** Characteristics of studies on VDR TaqI rs731236 (T/C) variant and polycystic ovarian syndrome (PCOS) susceptibility

Author	Year	Country	Ethnicity	NOS scores	Sample size		Genotype in cases			Genotype in controls		
					PCOS	Control	CC	CT	TT	CC	CT	TT
Dasgupta [14]	2015	India	Asian	7	252	401	47	92	113	105	110	186
Jedrzejuk [15]	2015	Poland	Caucasian	7	90	98	8	45	37	12	37	49
Mahmoudi [16]	2015	Iran	Asian	8	35	35	6	14	15	4	16	15
Mahmoudi [17]	2009	Iran	Asian	8	162	162	20	71	71	14	76	72
El-Shal [18]	2013	Egypt	Caucasian	8	150	150	36	74	40	20	61	69
Siddamalla [19]	2017	India	Asian	7	95	130	24	31	40	17	42	71
Bagheri [24]	2013	Iran	Asian	7	38	38	8	14	16	2	19	17
Wehr [20]	2011	Australia	Caucasian	7	536	137	72	238	226	23	65	49
Cao [21]	2016	China	Asian	7	120	120	11	52	57	8	72	40
Santos [22]	2018	Brazil	Caucasian	7	188	99	70	87	31	40	48	11

**Table 4:** Characteristics of studies on VDR FokI rs2228570 (C > T) variant and polycystic ovarian syndrome (PCOS) susceptibility

Author	Year	Country	Ethnicity	NOS scores	Sample size		Genotype in cases			Genotype in controls		
					PCOS	Control	TT	TC	CC	TT	TC	CC
Dasgupta [14]	2015	India	Asian	7	250	249	8	87	155	9	88	152
Jedrzejuk [15]	2015	Poland	Caucasian	7	90	98	11	51	28	25	50	23
Mahmoudi [16]	2015	Iran	Asian	8	35	35	2	17	16	1	10	24
Mahmoudi [17]	2009	Iran	Asian	8	162	162	12	67	83	7	59	96
Bagheri [23]	2012	Iran	Asian	7	46	46	22	20	4	29	15	2
Wehr [20]	2011	Australia	Caucasian	7	538	135	82	241	215	22	60	53
Cao [21]	2016	China	Asian	7	120	120	10	40	70	10	45	65

**Table 5:** Characteristics of studies on VDR TaqI rs757343 (G > A) variant and polycystic ovarian syndrome (PCOS) susceptibility

Author	Year	Country	Ethnicity	NOS scores	Sample size		Genotype in cases			Genotype in controls		
					PCOS	Control	AA	AG	GG	AA	AG	GG
Bagheri [23]	2012	Iran	Asian	7	181	181	7	51	123	6	48	127
Ranjzad [25]	2012	Iran	Asian	8	35	35	1	6	28	0	8	27
Zadeh-Vakili [26]	2013	Iran	Asian	8	221	260	6	58	157	8	66	186

### Meta-analysis results of *VDR ApaI rs7975232 (A > C)* variant and PCOS susceptibility

**Fig. 2:**
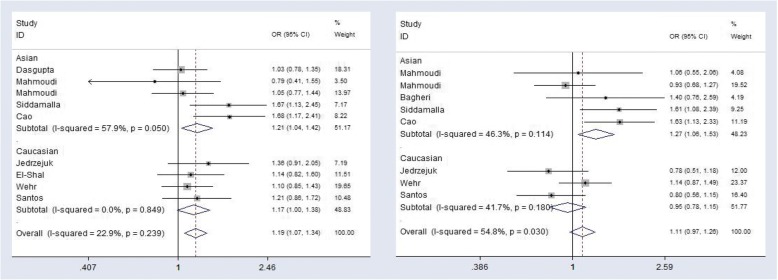
Forest plots of the VDR polymorphisms and PCOS in the overall populations. VDR ApaI (rs7975232) with allelic model: C vs. A; and VDR BsmI (rs1544410) with allelic model: G vs. A

The detailed results of the relationship between *VDR gene ApaI rs7975232 (A > C)* variant and PCOS susceptibility are shown in Table 6. A total of 9 studies on the relationship between VDR ApaI rs7975232 (A > C) variation and PCOS susceptibility were included. The heterogeneity test demonstrated no significant heterogeneity exist in all studies and the fixed effects model results on Mantel-Haenszel method were used. We found a significant association of the *VDR gene ApaI rs7975232 (A > C)* polymorphism with PCOS susceptibility in the allelic (C vs. A: OR = 1.19, 95%CI = 1.06~1.34, *P* = 0.004), recessive (CC + CA vs. AA: OR = 1.20, 95%CI = 1.01~1.42, *P* = 0.042) and dominant (CC vs. CA + AA: OR = 1.35, 95%CI = 1.09~1.69, *P* = 0.008) genetic models in the overall populations. Population subgroup analysis showed that there was a significant correlation between VDR ApaI rs7975232 (A > C) polymorphism and PCOS susceptibility in the Asian population (allelic model C vs. A: OR = 1.21, 95%CI = 1.04~1.42, *P* = 0.016; dominant model CC vs. CA + AA: OR = 1.70, 95%CI = 1.26~2.29, *P* = 0.001) (Fig. 2), but this association was not found in the Caucasian population.

**Table 6:** Summary of meta-analysis on VDR gene variants and polycystic ovarian syndrome (PCOS) susceptibility

Polymoyphisms	Population	Genetic model	Genetic model	No. of studies	Test of association			Model	Test of heterogeneity		Egger’s test(*P*)
					OR	95% CI	*P*-value		*P*-value	I^2^ (%)	
rs7975232	All	C vs. A	Allelic	9	1.19	1.06~1.34	0.004	F	0.170	32.5	0.676
VDR ApaI		CC vs. CA + AA	Dominant	9	1.35	1.09~1.69	0.008	F	0.310	16.0	0.145
		CC + CA vs. AA	Recessive	9	1.20	1.01~1.42	0.042	F	0.050	50.0	0.132
	Asian	C vs. A	Allelic	5	1.21	1.04~1.42	0.016	F	0.060	57.9	0.963
		CC vs. CA + AA	Dominant	5	1.70	1.26~2.29	0.001	F	0.700	0.0	0.265
		CC + CA vs. AA	Recessive	5	1.10	0.88~1.37	0.411	R	0.018	66.4	0.234
	Caucasian	C vs. A	Allelic	4	1.17	0.98~1.38	0.053	F	0.849	0.0	0.452
		CC vs. CA + AA	Dominant	4	1.11	0.83~1.48	0.470	F	0.578	0.0	0.145
		CC + CA vs. AA	Recessive	4	1.32	0.89~1.46	0.084	F	0.683	0.0	0.247
rs1544410	All	G vs. A	Allelic	8	1.11	0.91~1.37	0.307	R	0.030	54.8	0.462
VDR BsmI		GG vs. GA + AA	Dominant	8	0.94	0.75~1.17	0.556	F	0.280	20	0.563
		GG + GA vs. AA	Recessive	8	1.08	0.45~2.62	0.860	R	0.030	57	0.245
	Asian	G vs. A	Allelic	5	1.27	1.06~1.53	0.011	F	0.114	46.3	0.256
		GG vs. CA + AA	Dominant	5	1.89	1.08~3.30	0.026	R	0.027	63.5	0.356
		GG + GA vs. AA	Recessive	5	1.54	0.63~3.76	0.342	R	0.001	86.1	0.235
	Caucasian	G vs. A	Allelic	3	0.95	0.78~1.15	0.597	F	0.180	41.7	0.751
		GG vs. CA + AA	Dominant	3	0.57	0.27~1.18	0.128	R	0.002	84.3	0.156
		GG + GA vs. AA	Recessive	3	0.46	0.35~1.28	0.136	R	0.069	62.7	0.237
rs731236	All	C vs. T	Allelic	10	1.14	0.93~1.40	0.218	R	0.001	67.5	0.452
VDR TaqI		CC vs. TC + TT	Dominant	10	1.20	0.84~1.71	0.322	R	0.006	61.1	0.564
		CC + TC vs. TT	Recessive	10	1.07	0.81~1.41	0.628	R	0.003	63.5	0.521
	Asian	C vs. T	Allelic	6	1.09	0.85~1.41	0.493	R	0.034	58.6	0.426
		CC vs. CT + TT	Dominant	6	1.46	0.81~2.64	0.207	R	0.008	68.2	0.359
		CC + CT vs. TT	Recessive	6	1.02	0.83~1.24	0.858	F	0.128	41.6	0.215
	Caucasian	C vs. A	Allelic	4	1.19	0.82~1.74	0.359	R	0.002	79.5	0.568
		CC vs. CA + AA	Dominant	4	1.01	0.63~1.63	0.961	R	0.062	59.1	0.356
		CC + CA vs. AA	Recessive	4	1.15	0.63~2.10	0.654	R	0.001	80.8	0.628
rs2228570	All	T vs. C	Allelic	7	1.04	0.83~1.30	0.715	R	0.050	52.3	0.539
VDR FokI		TT vs. CT + CC	Dominant	7	0.90	0.65~1.24	0.521	F	0.291	18.1	0.759
		TT + CT vs. CC	Recessive	7	1.06	0.88~1.27	0.569	F	0.149	36.6	0.349
	Asian	T vs. C	Allelic	5	1.13	0.94~1.37	0.190	F	0.150	40.8	0.564
		TT vs. CT + CC	Dominant	5	1.26	0.76~2.08	0.374	F	0.785	0.0	0.486
		TT + CT vs. CC	Recessive	5	1.15	0.92~1.44	0.233	F	0.136	42.8	0.843
	Caucasian	C vs. A	Allelic	2	0.85	0.68~1.07	0.173	F	0.124	57.7	–
		CC vs. CA + AA	Dominant	2	0.65	0.29~1.44	0.284	R	0.084	66.5	–
		CC + CA vs. AA	Recessive	2	0.88	0.63~1.23	0.465	F	0.352	0.0	–
rs757343	All (Asian)	A vs. G	Allelic	3	1.04	0.81~1.34	0.734	F	0.939	0.0	0.428
VDR Tru9I		AA vs. AG + GG	Dominant	3	1.09	0.52~2.28	0.830	F	0.753	0.0	0.740
		AA+AG vs. GG	Recessive	3	1.05	0.79~1.39	0.759	F	0.899	0.0	0.445

*F* fixed effects model, *R* random effects model

### Meta-analysis results of *VDR BsmI rs1544410 (A > G)* variant and PCOS susceptibility

In Table 6, a total of 8 studies were included to study the relationship between polymorphism of *VDR BsmI rs1544410 (A > G)* and PCOS susceptibility. Significant heterogeneity was found in some comparisons and results from the random-effects model using the DerSimonian-Laird method were used. We did not find a correlation between *VDR BsmI rs1544410 (A > G)* polymorphism and PCOS susceptibility in all genetic models of the general population. Subgroup analysis by ethnicity revealed a significant association between polymorphism of *VDR BsmI rs1544410 (A > G)* and susceptibility to polycystic ovary syndrome in the Asian population (allelic model: G vs. A: OR = 1.27, 95%CI = 1.06~1.53, *P* = 0.011; dominant model: GG vs. CA + AA: OR = 1.89, 95%CI = 1.08~3.30, *P* = 0.026) (Fig. 2), but this association was not found in the Caucasian population.

### Meta-analysis results of *VDR TaqI rs731236 (T > C)* variant and PCOS susceptibility

In Table 6, 10 studies were included about the relationship between the *VDR TaqI rs731236 (T > C)* polymorphisms and PCOS susceptibility. Significant heterogeneity was found in most comparisons, and random effects model results on DerSimonian-Laird method were used. We found no correlation between variation and PCOS susceptibility in the general population and in subgroups by ethnicity.

### Meta-analysis results of *VDR FokI rs2228570 (C > T)* variant and PCOS susceptibility

Table 6 included 7 studies on the relationship between VDR FokI rs2228570 (C > T) variation and PCOS susceptibility.. The heterogeneity test demonstrated no significant heterogeneity exist in all studies and fixed effects model results on Mantel-Haenszel method were used. There is no association of the *VDR FokI rs2228570 (C > T*) variant with PCOS susceptibility was found in the overall population and sub-groups by ethnicity.

### Meta-analysis results of *VDR Tru9I rs757343 (G > A)* variant and PCOS susceptibility

In Table 6, a total of 3 studies examined the relationship between *VDR Tru9I rs757343 (G > A)* variation and PCOS susceptibility. The subjects of all included studies were conducted in the Asian populations. The heterogeneity test demonstrated no significant heterogeneity exist in all studies and fixed effects model results on Mantel-Haenszel method were used. There is no association between the *VDR Tru9I rs757343 (G > A)* polymorphism and PCOS susceptibility was found in the Asian population.

### Publication bias

**Fig. 3:**
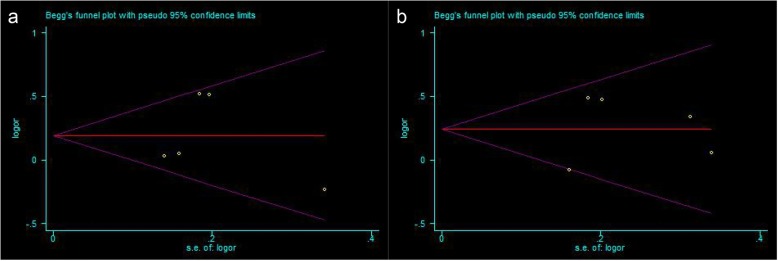
Begg’s funnel plot of the VDR polymorphisms and PCOS in the Asian population. **a** VDR ApaI (rs7975232) with allelic model: C vs. A; **b** VDR BsmI (rs1544410) with allelic model: G vs. A

Except for the polymorphism of VDR FokI rs2228570 (C > T) and the risk comparison of PCOS in all white genetic models, as these comparisons included only two studies, the Begg and Egger trials were carried out in all comparisons. Begg’s funnel plots were performed in all comparisons showed the shape was symmetrical, and the Egger’s linear regression analysis further indicated that there was no publication bias in the meta analysis (Table 6, Fig. 3).

### Sensitive analysis

**Fig. 4:**
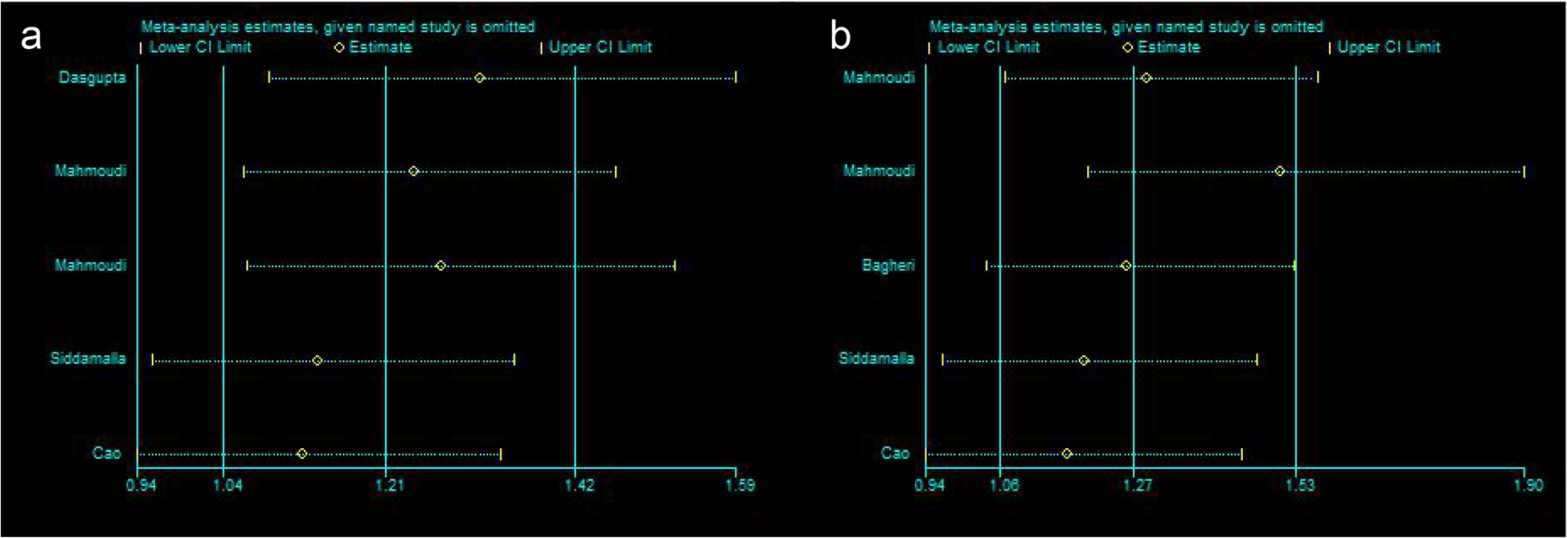
Sensitive analysis by omitting one study at a time to evaluate the stability of results. **a** VDR ApaI (rs7975232) with allelic model: C vs. A; **b** VDR BsmI (rs1544410) with allelic model: G vs. A

Sensitive analysis was conducted to estimate if our results were substantially affected by the presence of any individual. Our results suggest that no single study has a significant effect on the merger effect (Fig. 4).

## Discussion

Genetic factors have become increasingly important in the progression of PCOS. Previous studies have shown that *VDR* gene variants are associated with serum insulin levels in women with PCOS [33]. Serum 25-hydroxyvitamin D [25 (OH) D] has been shown to have a negative effect on *VDR*-mediated insulin resistance by regulating the expression of target genes [16]. *VDR* gene involved in insulin signaling pathway is considered as an important candidate gene for PCOS [34]. However, previous genome-wide association studies (GWAS) of Chinese patients with PCOS have not found VDR gene as a new susceptibility site for PCOS [35, 36]. Subsequently, Louwers et al. conducted a cross-racial meta-analysis on the genetic variation of pcos [37].The meta-analysis, which included data from Chinese, US, and Dutch data showed that 12 important variants were mapped to FSHR, LHCGR, SUMO1P1, YAP1, DENND1A, THADA, RAB5B/SUOX, c9orf3 loci, but not included VDR gene [37–39].

In this meta-analysis, we summarized the existing data on the associations of *VDR* polymorphisms and PCOS susceptibility from available databases. The results included a total of 13 articles based on 1922 PCOS patients and 1665 controls, showed that *VDR ApaI (rs7975232)* and *VDR BsmI (rs1544410)* polymorphisms are associated with PCOS susceptibility and *VDR TaqI (rs731236), VDR FokI (rs2228570), VDR Tru9I (rs757343)* did’t reveal a relationship with the PCOS susceptibility. The results were in accordance with previous studies and might provide a new biomarker in the etiology of PCOS [14, 18, 20]. We also performed a subgroup analysis to further explore the potential impact of patient ethnicity on the relationship between *VDR* polymorphisms and PCOS risk. Subgroup analysis by ethnicity showed that VDR ApaI (rs7975232) and VDR BsmI (rs1544410) polymorphisms were significantly correlated with PCOS susceptibility in the Asian population but not in the Caucasian population. The reason for this finding may be genetic disparities between the ethnic groups. Due to the process of natural selection, different groups might have some differences in the functional variants [40].

Accordingly, *VDR* gene polymorphism may play a role in the pathogenesis of PCOS by affecting the insulin signaling pathway [13]. However, since these polymorphisms are largely nonfunctional, it seems likely that linkage imbalances with another unknown functional variant of the *VDR* gene would explain the observed association. In addition, *VDR* gene polymorphism may play a role in the pathogenesis of PCOS by affecting the PTH-vitamin D axis [41]. Consistent with this view, *VDR* gene polymorphism is associated with serum PTH and 25 (OH) D levels, and vitamin D-VDR complex inhibits the secretion and synthesis of PTH [42]. Simsek et al. conducted a systematic review suggesting that vitamin D status is negatively associated with metabolic disorders in PCOS [43]. Next, he demonstrated that serum 25(OH)D was significantly lower in women with PCOS than in the birth control group. Poor lipids and a high HOMA-IRA were associated with vitamin D status in women with PCOS [44].

The heterogeneity was observed in some comparisons, but partially it was resolved by subgroup analysis based on ethnicity. Our research found that VDR can act as an influencing factor on PCOS. These SNP mutations can be used as risk factors to evaluate PCOS.The results of this meta-analysis were different from those of Han et al. ‘s previous meta-analysis [27], which showed that *VDR* gene polymorphism in *TaqI (rs731236)* for T allele was significant association with PCOS and didn’t find any association between *VDR ApaI (rs7975232), VDR BsmI (rs1544410), VDR FokI (rs2228570), VDR Tru9I (rs757343)* and PCOS susceptibility in the all included studies. Such inconsistent results may be due to different number of studies included in the meta-analysis, different sample sizes and different statistical abilities. In their study, they conducted a meta-analysis and included 5 (9 studies in the present meta) studies on *VDR ApaI rs7975232 (G > T)*, 4 (8 studies in the present meta) studies on *VDR BsmI rs1544410 (A > G)*, 6 (10 studies in the present meta) studies on *VDR TaqI rs731236 (T > C)*, 5 (7 studies in the present meta) studies on *VDR FokI rs2228570 (C > T)* and didn’t include *VDR Tru9I rs757343 (G > A)* studies (3 in the present meta) for analysis the association with PCOS susceptibility. In addition, we conducted a subgroup analysis by ethnicity among Asian and Caucasian populations, which was not included in their meta-analysis.

Although the present meta-analysis has the advantage of a relatively large sample size for a combined result, several limitations should be addressed in interpreting our results. Firstly, we included relevant articles published only in English and Chinese so that potential language bias may exist in this study. Second, most of the studies were conducted in Asian populations, and the small number of studies in the Caucasian subgroup analysis may have resulted in insufficient statistical ability to detect subtle relationships. Third, age, gender, genetic variation, environmental factors exposure and other risk factors may have an impact on the incidence of PCOS, but this study only considered gene polymorphism. The effects of gene-gene and gene-environment interaction on the occurrence and development of the disease need to be further studied.

## Conclusions

In summary, current meta-analysis provided statistical evidence that *VDR ApaI (rs7975232)* and *VDR BsmI (rs1544410)* polymorphisms are associated with PCOS susceptibility in the Asian population and *VDR TaqI (rs731236), VDR FokI (rs2228570), VDR Tru9I (rs757343)* did’t reveal a relationship with the PCOS susceptibility. These results might not be generalized to other ethnic populations. Further studies with more sample size and including other confounding factors are still needed in the future for a definitive conclusion.

## Abbreviations

95% CIs: 95% confidence intervals
ORs: Odds ratios
PCOS: Polycystic ovary syndrome
SNP: Single nucleotide polymorphism
VDR: Vitamin D receptor
